# Exploring Central Vascular Anatomy With Axial Computed Tomography During Surgery for Sigmoid Colon and Rectal Cancer: New Insights Into the Anatomical Relationship Between the Inferior Mesenteric Artery and the Duodenum

**DOI:** 10.3389/fsurg.2021.785313

**Published:** 2021-12-13

**Authors:** Zhiqiang Cheng, Pengfei Ren, Xiaoyan Wang, Kexin Wang, Zhibo Yan, Dongsong Bi, Yanlei Wang, Yong Dai, Xiang Zhang

**Affiliations:** ^1^Department of General Surgery, Qilu Hospital of Shandong University, Jinan, China; ^2^Department of General Surgery, Lingcheng People's Hospital, Dezhou, China; ^3^Department of Neonatology, Weifang Yidu Central Hospital, Weifang, China

**Keywords:** colorectal surgery, inferior mesenteric artery, duodenum, lymph node dissection, computed tomography

## Abstract

**Background:** In some individuals, the inferior mesenteric artery (IMA) originates from the aorta above the lower edge of the duodenum. This anatomical feature has rarely been reported but may be important in guiding central vascular ligation and lymph node dissection in colorectal surgery. This retrospective study aimed to explore the anatomical relationship between the IMA and the duodenum and evaluate its potential impact on the efficacy of D3 lymph node dissection.

**Methods:** A total of 439 patients undergoing laparoscopic colorectal surgery at the Department of General Surgery, Qilu Hospital of Shandong University, were retrospectively enrolled. Clinical data from axial computed tomography (CT) scans were collected and analysed.

**Results:** In 27.69% of patients, the IMA originated at or above the lower edge of the duodenum (median distance: −8 mm). These patients were characterised by a shorter superior mesenteric artery to aortic bifurcation distance, a superiorly located IMA origin, and a greater distance between the IMA and both the left colic artery and the inferior mesenteric vein. The number of harvested lymph nodes was not significantly associated with the distance between the IMA and the duodenum (*P* = 0.858).

**Conclusions:** Preoperative axial CT scans can provide a great deal of information regarding central vascular anatomy in the context of sigmoid colon and rectal cancer surgery. Nearly one-third of patients have the IMA originating at or above the duodenum. Whether this anatomical feature affects D3 lymph node dissection warrants further investigation.

## Introduction

Surgical resection is the most effective treatment for colorectal cancer in the absence of distal metastasis. Besides resection of the primary tumour with sufficient margins, complete removal of the regional lymph nodes also contributes to decreased cancer recurrence and better prognosis by eradicating otherwise undetected tumour cells and by improving the accuracy of lymph node evaluation. The Japanese concept of D3 dissection, which is similar to the Western concept of central vascular ligation with extensive lymphadenectomy, is recommended for sigmoid colon and rectal cancer if lymph node metastasis is suspected, or if the clinical depth of tumour invasion (cT) is stage II or higher (1). According to the Japanese Society for Cancer of the Colon and Rectum (JSCCR) (2), the D3 approach requires dissection of the pericolic/perirectal, intermediate, and main lymph nodes. The main lymph nodes in cancer of the sigmoid colon and rectum are also known as inferior mesenteric root nodes or No. 253 lymph nodes, referring to the lymph nodes along the inferior mesenteric artery (IMA), from the origin of the IMA to the origin of the left colic artery (LCA). In surgical practise, the IMA is usually resected at 1.0–1.5 cm distal to its origin, after radical dissection of the No. 253 lymph nodes, and the aorta is not routinely exposed to avoid autonomic nerve injury. However, the anatomical relationship between the root of the IMA and the duodenum is variable (3). In some patients, the IMA originates from the aorta above the lower edge of the duodenum, and the root of the IMA may not be adequately exposed without mobilising the third segment of the duodenum. This anatomical variation has rarely been reported but may be of great significance in guiding surgical practise. Without knowledge of the presence of this anatomical feature, there is a possibility that the eradication ability of D3 dissection is compromised, leading to an unfavourable prognosis. In the present study, for the first time, we used computed tomography (CT) to retrospectively explore the central vascular anatomy during surgery for sigmoid colon and rectal cancer, and evaluated the anatomical relationship between the IMA and the lower edge of the duodenum, alongside other related aspects of the central vascular anatomy.

## Materials and Methods

### Patient Selection

A total of 439 Chinese patients (266 men and 173 women) undergoing laparoscopic colorectal surgery between January 2016 and October 2020 at the Department of General Surgery, Qilu Hospital of Shandong University, were retrospectively enrolled in the study. The inclusion criteria were as follows: (1) undergoing laparoscopic radical sigmoidectomy or proctectomy (including low anterior resection [AR] and abdominoperineal resection [APR] with D3 dissection) due to sigmoid colon or rectal adenomatous carcinoma; (2) contrast-enhanced abdominopelvic CT scan (1 mm slice) performed prior to surgery. The exclusion criteria were as follows: (1) previous abdominal surgery; (2) inability to remain in supine during CT scan; (3) signs of severe abdominal tortuous aorta; (4) signs of distant metastasis; (5) signs of bowel obstruction; and (6) neoadjuvant therapy. The study was approved by the Ethics Committee of Qilu Hospital of Shandong University.

### Contrast-Enhanced CT Scan Protocol

Contrast-enhanced CT scans were performed using multidetector row CT scanners 23 (Discovery CT750HD, GE Healthcare). The scanning parameters were as follows: patient in supine; abdominal and pelvic scanning range; 1 mm slice thickness; 120 kV tube voltage; 250–400 mA tube current using automatic tube current modulation; and a 512 × 512 matrix. Patients were injected with 1.5 mL/kg of iopromide (Ultravist 300; Bayer) at a rate of 3.0 mL/s via the antecubital vein through a power injector.

### Measurements

All measurements were performed in the arterial phase in the axial view on computers without three-dimensional (3D) reconstruction. The distance between the IMA origin and the lower edge of the duodenum (IMA–D) was measured at a virtual sagittal plane passing the IMA origin, and the value was recorded as negative if the IMA origin was located above the lower edge of the duodenum. The distance between the IMA origin and the umbilicus was measured as the distance between the IMA projection at the anterior body surface and the centre of the umbilicus. The distance between the IMA origin and the LCA origin or sigmoid artery (SA) origin was calculated using the Pythagorean theorem by measuring the horizontal and vertical distances from the branching location to the IMA origin. The length of the IMA was measured from its origin at the aorta to its first branch (i.e. LCA or SA). The distance between the LCA and the inferior mesenteric vein (IMV), as well as the distance between the IMA and the IMV, was determined at the level of the IMA origin. More specifically, the distance between the LCA and the IMV was determined either at the level of the LCA branching or at the level of the IMA origin, as appropriate.

### Statistical Analysis

Normally distributed continuous data are expressed as mean ± standard deviation and were analysed using Student's *t*-test; non-normally distributed data are expressed as median with 25 and 75% quartiles and were analysed using the Wilcoxon rank-sum test. Spearman's rank correlation test was used to analyse the degree of correlation between two variables. Categorical data were compared using the chi-square test or Fisher's exact test. All analyses were performed using IBM SPSS version 25.0 (IBMCorp., Armonk, NY, USA); *P* < 0.05 was considered statistically significant.

## Results

### Baseline Characteristics

The median age of the enrolled patients was 60 years (25 and 75% quartiles: 52, 67 years). Height was recorded only in 279 patients, of which 164 were men and 115 were women. The median height was 165 (160, 170) cm, and the median body mass index was 24.5 (22.5, 26.6) kg/m^2^. Laparoscopic sigmoidectomy/AR was performed in 361 patients, while laparoscopic APR was performed in 78 patients. The median duration of surgery was 180 (150, 225) min. The number of patients classified into TNM stages I, II, and III, according to the eighth edition of the American Joint Committee on Cancer TNM staging system (4), were 27, 239, and 173, respectively (Table 1). Thirty-six patients with rectal cancer underwent neoadjuvant radiotherapy with capecitabine monotherapy.

**Table 1:** Baseline characteristics.

	**Total**	**Male**	**Female**
Patient number	439	266	173
Age (years)	60 (52–67)	64 (52–66)	61 (52–67)
Height (cm)^*^^#^	165 (160–170)	170 (166–173)	160 (156–163)
BMI (kg/m^2^)^#^	24.5 (22.5–26.6)	24.5 (22.7–26.6)	24.5 (22.4–26.6)
**Surgery**			
laparoscopic sigmoidectomy/AR	362	219	143
laparoscopic APR	77	47	30
Time duration of surgery (min)	180 (150–225)	180 (150–230)	180 (150–215)
Neoadjuvant chemoradiotherapy	36	24	12
**TNM stage** ^&^			
I	18	9	9
II	243	154	89
III	178	103	75

* *Men vs. women, P < 0.05*.

# *Height and BMI were only recorded in 279 patients*.

& *According to the eighth edition of the AJCC TNM system*.

### Location of the IMA Origin

The IMA origin was located below the superior mesenteric artery (SMA) origin and above the aortic bifurcation, with the median SMA–IMA and IMA–aortic bifurcation distances being 62 (56, 74) mm and 37 (32, 43) mm, respectively. The projection of the IMA origin at the anterior body surface was above the umbilicus, with a median distance of 34 (25, 47) mm. At the IMA origin level, the median IMA–IMV distance was 24 (19, 30) mm. The length of the IMA was 32 (26.6, 30.6) mm.

### Distance Between the IMA Origin and the Lower Edge of the Duodenum (IMA–D)

**Figure 1:**
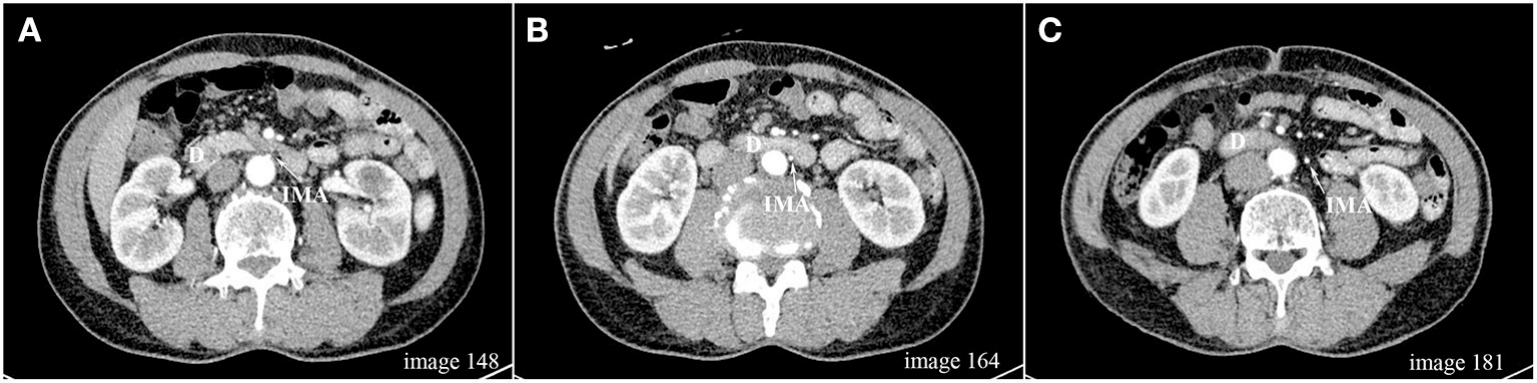
**(A–C)** Axial scan images (1 mm slice) from one patient demonstrating that the IMA origin located above the lower edge of the duodenum; D, duodenum; IMA, inferior mesenteric artery.

In two patients, the lower edge of the duodenum and the duodenojejunal flexure did not cross the virtual sagittal plane passing the IMA origin, and they were therefore excluded from this analysis. In the remaining 437 patients, the median IMA–D distance was 9 (−1, 22.5) mm. The IMA origin was located at or above the lower edge of the duodenum (IMA–D≤0) in 121 patients (27.69%), with a median distance of −8 (−3, −13) mm (Figure 1), while the IMA origin was located below the lower edge of the duodenum (IMA–D>0) in 316 patients (72.31%), with a median distance of 16 (8, 26) mm.

### Subgroup Analysis Between IMA–D ≤ 0 and IMA–D > 0 Patients

In the 121 patients with IMA–D ≤ 0, the IMA origin was closer to the SMA origin (*P* < 0.001) and further away from the aortic bifurcation (*P* = 0.014), with greater IMA–LCA (*P* = 0.001) and IMA–SA (*P* < 0.001) distances, than patients with IMA–D > 0. The distance between the SMA origin and the aortic bifurcation was shorter in patients with IMA–D ≤ 0 than in those with IMA–D > 0 (*P* = 0.003), even though the height of the patients was comparable between the two groups (*P* = 0.222). The IMA–IMV and LCA–IMV distances (the latter at the LCA origin) were both greater in patients with IMA–D ≤ 0 than in those with IMA–D > 0 (*P* = 0.015 and *P* = 0.002, respectively). This indicates that patients with IMA–D ≤ 0 were characterised by a shorter SMA–aortic bifurcation distance, a more superiorly located IMA origin, and greater IMA–LCA, IMA–SA, IMA–IMV, and LCA–IMV distances, while still being of comparable height to patients with IMA–D > 0 (Table 2).

**Table 2:** Patients with the IMA locating at or above the duodenum vs. below the duodenum.

	**Total**	**IMA at or above the duodenum** **(IMA-D ≤ 0)**	**IMA below the duodenum (IMA-D > 0)**	***P* value**
IMA-D (mm)^*^	9 (−1, 22.5)	−8 (−3, −13)	16 (8, 26)	N/A
SMA-IMA (mm)^*^	62 (56, 74)	58 (52, 66)	65 (58, 76)	<0.001
SMA-Aortic Bifurcation (mm)^*^	98 (90, 115)	95 (88, 111)	100 (91, 118)	0.003
IMA-Aortic Bifurcation (mm)^*^	37 (32, 43)	38 (33, 44.5)	36 (31, 42)	0.014
IMA-Umbilicus (mm)^*^	34 (25, 47)	38 (27.5, 51.5)	32 (23, 44.25)	0.003
IMA-IMV (mm)^*^	24 (19, 30)	26 (20, 31.5)	24 (19, 29)	0.015
IMA-LCA (mm)^*^	32 (26.6, 30.6)	36.1 (28.2, 42)	31.4 (26, 39)	0.001
IMA-SA (mm)^*^	38.6 (32.2, 47.9)	42.1(36.1, 49.9)	37.2(31.6, 47.0)	<0.001
LCA-D (mm)^*^	40 (29, 53)	25 (17, 34)	46.5 (35, 58)	<0.001
SMA-D (mm)^*^	54 (44, 66)	66 (59.5, 79)	50 (41, 59)	<0.001
LCA-IMV at the IMA origin (mm)^&^	29 (15, 48)	28 (14.5, 48)	29 (15, 48)	0.954
LCA-IMV at the LCA origin (mm)^*^	13 (11, 15)	13 (12, 16)	13 (11, 15)	0.002
Height (cm)^#^	165 (160–170)	165 (160, 170)	166 (160, 171.5)	0.222
Time duration of surgery (min)	180 (150–225)	178 (147.5, 227.5)	180 (150, 221.3)	0.829
Number of lymph nodes harvested	16 (14, 20)	16 (14, 21)	16 (14, 20)	0.858
**Sex**				
Men	265	69 (57.02%)	196 (62.03%)	0.338
Women	172	52 (42.98%)	120 (37.97%)	
**Branching types of the IMA^%^**				
Type 1	238 (54.46%)	66 (54.55%)	172 (54.43%)	0.338
Type 2	93 (21.28%)	29 (23.97%)	64 (20.25%)	
Type 3	104 (23.80%)	25 (20.66%)	79 (25%)	
Type 4	2 (0.46%)	1 (0.82%)	1 (0.32%)	

# *Height was only recorded in 279 patients*.

* *Patients with IMA-D ≤ 0 vs. patients IMA-D > 0, P < 0.05*.

& *Calculated based on data from the 106 patients classified as type B*.

% *Calculated based on data from the 437 patients included for IMA-D analysis*.

### Associations Between IMA–D and Lymph Node Dissection

In the 437 patients included in the IMA–D analysis, the median number of harvested lymph nodes was 16 (14, 20). For patients with IMA–D ≤ 0 and IMA–D > 0, the median number of harvested lymph nodes was 16 (14, 21) and 16 (14, 20), respectively. No significant association between IMA–D distance and the number of harvested lymph nodes was found (*P* = 0.858). When considering only stage II and III patients, there remained no significant association between IMA–D distance and the number of harvested lymph nodes (*P* = 0.906). Eight patients with IMA–D ≤ 0 and 28 patients with IMA–D > 0 underwent neoadjuvant radiotherapy with capecitabine monotherapy. After excluding these patients, the number of harvested lymph nodes was 16 (14, 21) and 17 (14, 20) for IMA–D ≤ 0 and IMA–D > 0, respectively, which did not show any significant difference (*P* = 0.848).

### IMA Branching Types

IMA branching was categorised into four types based on a previously reported definition (5), with minor modifications, as follows: (1) Type 1, the LCA and SA arose independently from the IMA; (2) Type 2, the IMA and SA had a common trunk >5 mm in length; (3) Type 3, the LCA and SA arose from the IMA at the same point, or had a common trunk no >5 mm; and (4) Type 4, the LCA was lacking. Among all 439 patients, the majority were classified as Type 1 (*n* = 239, 54.44%). The number of patients classified as Type 2 or 3 was 93 (21.18%) and 105 (23.92%), respectively. It was extremely rare for the LCA to be lacking, with only 2 patients (1 man and 1 woman, 0.46%) classified as Type 4. The median distance between the IMA and LCA origins was 32 (26.6, 40.6) mm, and the median distance between the IMA and SA origins was 38.6 (32.2, 47.9) mm.

### LCA and IMV Organisation at the Level of the IMA Origin

**Figure 2:**
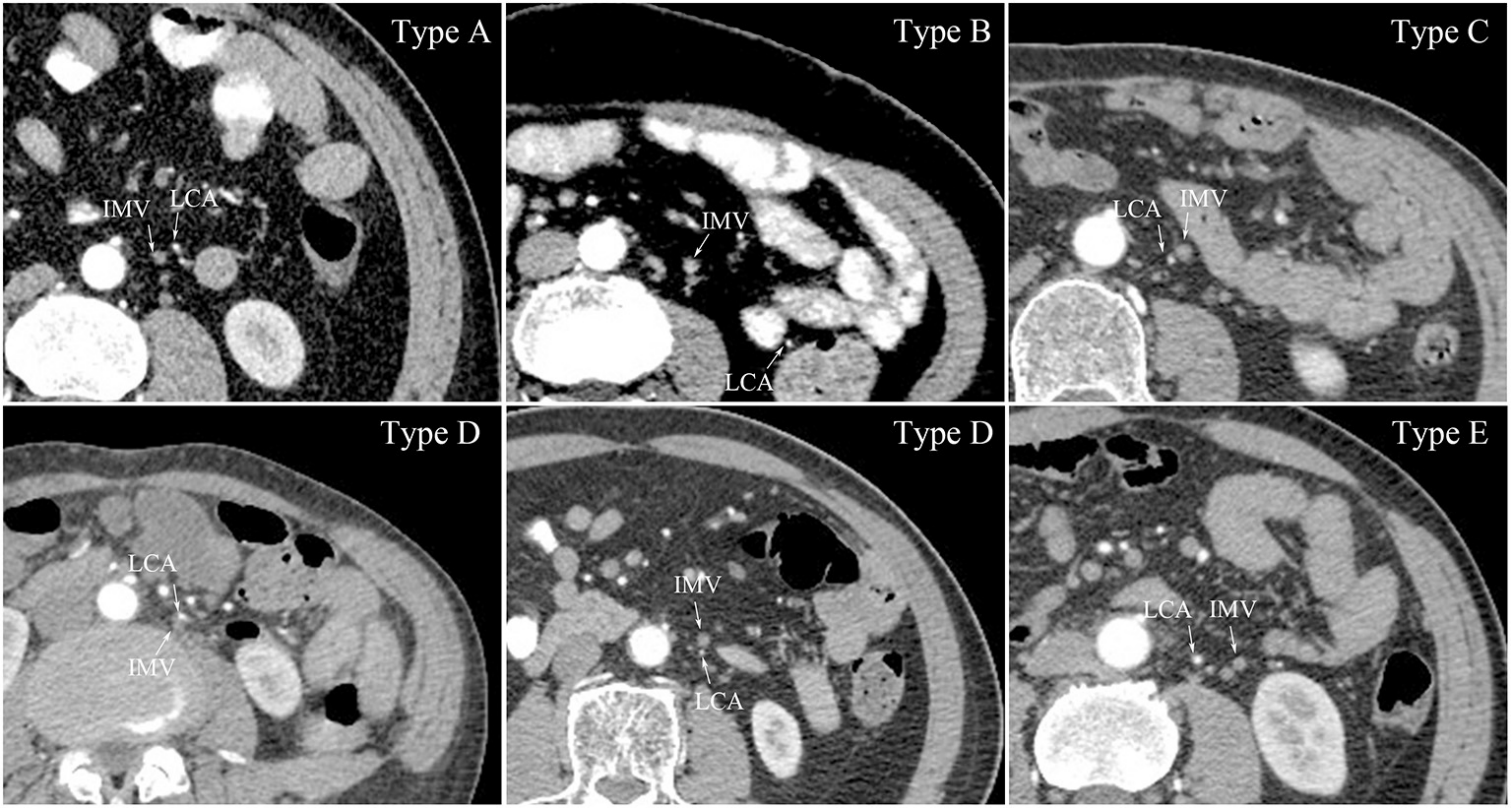
LCA and IMV organisation at the level of the IMA origin. Type A, the LCA located just lateral to the IMV; Type B, the LCA located laterally distant from the IMV; Type C, the LCA located just medial to the IMV; Type D, the LCA located either anterior or posterior to the IMV; Type E, the LCA located medially distant from the IMV; LCA, left colic artery; IMV, inferior mesenteric vein.

LCA and IMV organisation was categorised into five types (Figure 2), as follows: (1) Type A, the LCA was located just laterally to the IMV; (2) Type B, the LCA was located laterally at a distance from the IMV (more than 10 mm); (3) Type C, the LCA was located just medially to the IMV; (4) Type D, the LCA was located either anteriorly or posteriorly to the IMV; and (5) Type E, the LCA was located medially at a distance from the IMV (more than 10 mm). In the 437 patients not lacking the LCA, Type A was the most frequent organisational pattern (*n* = 145, 33.18%). The number of patients classified as Type B was 106 (24.26%), with a median distance of 29 (15, 48) mm. Type C and Type D included 68 patients (15.56%) and 115 patients (26.32%), ‘respectively. Only 3 patients (0.68%) had the LCA located medially at a distance from the IMV, with distances of 11 mm, 16 mm, and 17 mm. For the crossing type, the LCA ran ventrally across the IMV in 322 patients (73.68%), and dorsally in 115 patients (26.32%). The median distance between the LCA and IMV at the level of the LCA origin was 13 (11, 15) mm.

### Subgroup Analysis Between Men and Women

The median height of men was greater than that of women, as expected (*P* < 0.001). The SMA–IMA and SMA–aortic bifurcation distances were greater in men than in women (*P* = 0.008 and *P* < 0.001, respectively), and the projection of the IMA at the anterior body surface was also closer to the umbilicus in men than in women (*P* < 0.001). In correlational analysis, height was positively correlated with SMA–IMA distance (*P* = 0.026). At the level of the LCA origin, the LCA–IMV distance was greater in men than in women (*P* = 0.035). Concerning IMA branching type, there were fewer Type 2 and more Type 3 branching types in men than women (*P* = 0.024) (Table 3).

**Table 3:** Men vs. women.

	**Total**	**Male**	**Female**	***P* value**
SMA-IMA (mm)^*^	62 (56, 74)	65 (57, 76)	61 (54, 72)	0.008
SMA-Aortic Bifurcation (mm)^*^	98 (90, 115)	100 (93, 118)	94 (88, 113.5)	<0.001
IMA-Aortic Bifurcation (mm)	37 (32, 43)	37 (33, 44)	36 (31, 42)	0.05
IMA-Umbilicus (mm)^*^	34 (25, 47)	32 (22.8, 41)	41 (27, 53)	<0.001
IMA-IMV (mm)	24 (19, 30)	24 (20, 31)	23 (19, 28)	0.054
IMA-D (mm)^%^	9 (−1, 22.5)	9 (0, 23)	9.5 (-2, 22)	0.828
IMA-D ≤ 0 (*n*, %)	121 (27.69%)	69 (26.04%)	52 (30.23%)	0.338
IMA-D > 0 (*n*, %)	316 (72.31%)	196 (73.96%)	120 (69.77%)	
IMA-LCA (mm)	32 (26.6, 40.6)	32 (26.4, 39.6)	32.8 (26.8, 42.1)	0.091
IMA-SA (mm)	38.6 (32.2, 47.9)	38.5 (32, 47.1)	38.9 (33.4, 49)	0.179
LCA-D (mm)	40 (29, 53)	40 (28, 52)	42 (29, 55)	0.297
SMA-D (mm)	54 (44, 66)	56 (46, 66.5)	52 (42, 66)	0.054
LCA-IMV at the IMA origin (mm)^&^	29 (15, 48)	31 (15, 49)	26.5 (13.3, 45)	0.341
LCA-IMV at the LCA origin (mm)^*^	13 (11, 15)	13 (11, 16)	13 (10, 15)	0.035
Height (cm)	165 (160–170)	170 (166–173)	160 (156–163)	<0.001
Time duration of surgery (min)	180 (150, 225)	180 (150, 230)	180 (150, 215)	0.431
Number of lymph nodes harvested	16 (14, 20)	17 (14, 20)	16 (14, 20)	0.477
Branching types of the IMA^*^				
Type 1	239 (54.44%)	147 (55.26%)	92 (53.18%)	0.024
Type 2	93 (21.18%)	45 (16.92%)	48 (27.75%)	
Type 3	105 (23.92%)	73 (27.44%)	32 (18.50%)	
Type 4	2 (0.46%)	1 (0.38%)	1 (0.58%)	

* *Men vs. women, P < 0.05*.

& *Calculated based on data from the 106 patients classified as type B*.

% *Calculated based on data from the 437 patients included for IMA-D analysis*.

## Discussion

In the present study, we explored the anatomical relationship between the IMA and the lower edge of the duodenum, and reviewed the central vascular anatomy in the context of root lymph node dissection during surgery for sigmoid colon and rectal cancer. A great deal of information was successfully acquired using axial CT scans without 3D reconstruction, which represented a novel and economical approach. The key findings were as follows: (1) in 27.69% of patients, the IMA origin was located at or above the lower edge of the duodenum, and these patients were characterised by a shorter SMA–aortic bifurcation distance, a more superiorly located IMA origin, a longer IMA, and a greater IMA–IMV distance; (2) the number of harvested lymph nodes did not appear to be influenced by the anatomical relationship between the IMA and the duodenum; and (3) the definitions and outcomes of IMA branching types and LCA and IMV organisation were updated, in order that more detailed information to support preoperative preparation can be attained.

The anatomy of the IMA has been reported in previous studies and in anatomy textbooks. However, to our knowledge, the relationship between the IMA origin and the lower edge of the duodenum has only been reported in one study in recent decades (3). The authors measured IMA–D distance in 30 fresh cadavers and concluded that the IMA origin was located above the lower edge of the duodenum in 30% of cases, which is consistent with the results of the present study (27.69%). However, we note that the criteria used in the previous study were different. After revaluating the data reported in the previous study using the criteria of the present study, 20 of the 30 cases (67%) had the IMA origin located at or above the lower edge of the duodenum. This result is consistent with neither the results in the present study nor with our intuitive sense as developed during surgery. Therefore, a type II error cannot be ruled out in the previous report. Our conclusion was based on a relatively large cohort and objective CT scans. However, one limitation should be noted. The duodenum is a muscular organ, and the level of the lower edge varies with the state of contraction or dilation. A single CT scan cannot rule out the possibility that duodenal dilation leads to overlap of the IMA origin and the lower edge of the duodenum. Therefore, the true incidence of patients with the IMA origin located at or above the lower edge of the duodenum may be even less. To address this, we attempted to calculate an average IMA–D distance by adding data from a second CT scan performed during postoperative follow-up. However, the IMA origin was not visible in the follow-up CT scans of most patients, even in patients with a preserved LCA, and some patients were in any case lost during follow-up.

Main lymph node metastasis has been reported to occur in approximately 1.7–13.5% of sigmoid colon and rectal cancers (6–8). Despite this relatively low incidence, D3 dissection has been shown to convey a significant survival advantage in pT3 and pT4 colorectal cancer patients (9, 10). Additionally, D3 dissection can be safely performed with preservation of the LCA (“low tie”) and autonomic nerves via a laparoscopic approach (11, 12). The present study, for the first time, hypothesised that variation in the anatomical relationship between the IMA origin and the lower edge of the duodenum may have an impact on the efficacy of D3 dissection. Nevertheless, no difference in the total number of harvested lymph nodes was found, according to the location of the IMA origin relative to the lower edge of the duodenum. We are not however surprised by this result, because we had not identified this anatomical variation when performing the surgery. Moreover, lymph nodes were not assigned to the appropriate station and labelled for pathological assessment, as is standard practise in Japan (6), so neither the number nor the positivity of the No. lymph nodes could be directly compared. Therefore, additional well-designed randomised controlled studies are needed to adequately answer our primary hypothesis.

IMA branching type is important information in guiding sigmoid colon and rectal cancer surgery, particularly when both D3 dissection and preservation of the LCA are required. Variation in IMA branching type has historically been investigated by dissecting cadavers (13), using 3D CT angiography (14), or by intraoperative measurement (15). The unanimous conclusion was that the majority of patients had the LCA and the SA arising independently from the IMA (Type 1, 41.2–58%) and that a lack of LCA was rare (Type 4, 0.46–5.1%) (5, 14, 16). However, major inconsistencies lay in the incidence of Type 2 and Type 3 IMA branching types. After reviewing the literature, we found one explanation for this discrepancy: the Type 2 and Type 3 criteria had not been clearly defined, and were even contradictory between reports (5, 16). The criteria used in the present study were based on the most commonly reported definition, with minor modifications. An LCA and SA arising from the IMA at the same point and having a common trunk no >5 mm were both classified as Type 3, since a common trunk no >5 mm is not likely to be distinguishable. In the subgroup analysis, Type 3 was more common in men than in women, which was a novel discovery.

We also redefined the criteria for categorising LCA and IMV organisation at the level of the IMA origin. The LCA was located laterally to the IMV, either closely or distally, in more than half of the patients (Type A and Type B). In Types A, C, and D, the LCA was located close to the IMV; therefore, surgeons should exercise caution when dissecting the IMV. Type E was the least common type, with an incidence of only 0.68%. Despite this extremely low incidence, Type E suggests that surgical dissection is safe with a low probability of encountering the LCA within 24 mm (the median distance between the IMA and IMV) laterally of the IMA.

Several limitations of this study should be noted. First, as mentioned previously, a CT scan cannot rule out the influence of duodenal dilation; thus, prospective intraoperative validation is needed. Second, follow-up survival data were not available in the present study. Therefore, the potential hazards associated with incomplete D3 dissection in patients with the IMA located at or above the lower edge of the duodenum could not be further explored. Third, height was only reported in a limited number of patients, and correlational analysis between height and other parameters may therefore have been compromised.

In conclusion, preoperative axial CT scans can provide a great deal of information regarding central vascular anatomy, which can enhance preoperative preparation in sigmoid colon and rectal cancer surgery. In 27.69% of patients in this study, the IMA origin was located at or above the lower edge of the duodenum. Whether the presence of this anatomical feature affects the efficacy of D3 lymph node dissection warrants further investigation.

## Data Availability Statement

The raw data supporting the conclusions of this article will be made available by the authors, without undue reservation.

## Ethics Statement

The studies involving human participants were reviewed and approved by the Ethics Committee of Qilu Hospital of Shandong University. The patients/participants provided their written informed consent to participate in this study.

## Author Contributions

ZC, PR, XW, and XZ were involved in data collection and writing of the manuscript. YD, ZY, KW, DB, and XZ involved in conception. YW and XZ was involved in conception, design, and coordination of the study. PR, XW, and ZY were involved in picture and table formatting. YD and XZ are the guarantors of this work. All authors have critically reviewed the manuscript and have approved the publication of this final version of the manuscript.

## Funding

This study was supported by Clinical Practical and New Technology Development Fund of Qilu Hospital of Shandong University (2019-4).

## Conflict of Interest

The authors declare that the research was conducted in the absence of any commercial or financial relationships that could be construed as a potential conflict of interest.

## Publisher's Note

All claims expressed in this article are solely those of the authors and do not necessarily represent those of their affiliated organizations, or those of the publisher, the editors and the reviewers. Any product that may be evaluated in this article, or claim that may be made by its manufacturer, is not guaranteed or endorsed by the publisher.
